# Nanobiosensors as new diagnostic tools for SARS, MERS and COVID-19: from past to perspectives

**DOI:** 10.1007/s00604-020-04615-x

**Published:** 2020-11-05

**Authors:** Riccarda Antiochia

**Affiliations:** Department of Chemistry and Drug Technologies, Sapienza University of Rome, P.le Aldo Moro 5, 00185 Rome, Italy

**Keywords:** Nanobiosensor, Coronavirus, SARS, MERS, COVID-19, Point-of-care diagnostics, Immunosensor, DNA-sensor, Nanomaterial

## Abstract

The severe acute respiratory syndrome (SARS), Middle East respiratory syndrome (MERS) and novel coronavirus 19 (COVID-19) epidemics represent the biggest global health threats in the last two decades. These infections manifest as bronchitis, pneumonia or severe, sometimes fatal, respiratory illness. The novel coronavirus seems to be associated with milder infections but it has spread globally more rapidly becoming a pandemic. This review summarises the state of the art of nanotechnology-based affinity biosensors for SARS, MERS and COVID-19 detection. The nanobiosensors are antibody- or DNA-based biosensors with electrochemical, optical or FET-based transduction. Various kinds of nanomaterials, such as metal nanoparticles, nanowires and graphene, have been merged to the affinity biosensors to enhance their analytical performances. The advantages of the use of the nanomaterials are highlighted, and the results compared with those obtained using non-nanostructured biosensors. A critical comparison with conventional methods, such as RT-PCR and ELISA, is also reported. It is hoped that this review will provide interesting information for the future development of new reliable nano-based platforms for point-of-care diagnostic devices for COVID-19 prevention and control.

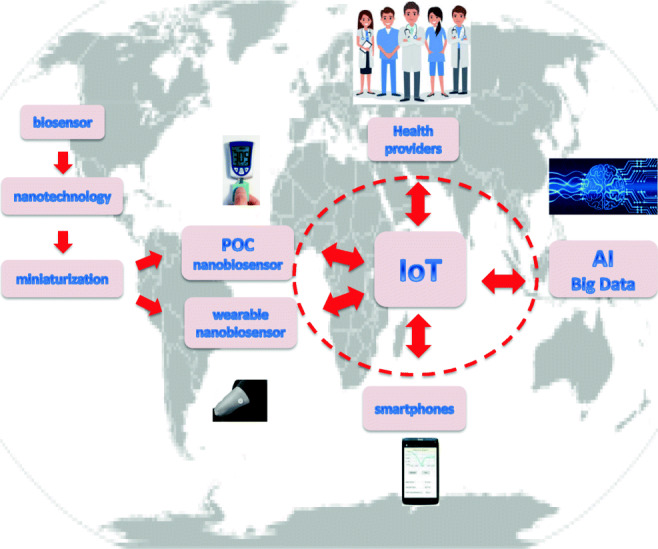

## Introduction

In the last two decades, we have witnessed the outbreak of three zoonotic, highly pathogenic human coronaviruses: severe acute respiratory syndrome coronavirus (SARS-CoV), Middle East respiratory syndrome coronavirus (MERS-CoV) and the 2019 novel coronavirus (2019-nCoV), named as severe acute respiratory syndrome coronavirus 2 (SARS-CoV-2) by the International Committee of Taxonomy of Viruses (ICTV) [1–3].

Coronaviruses are a large family of enveloped, single-stranded, positive-sense RNA viruses that mostly infect animals, such as birds and mammals, but may “spill over” from the animal host to human populations. There are seven coronaviruses infecting humans, four of them cause mild infection in the upper respiratory tract, whereas three of them (SARS-CoV, MERS-CoV, SARS-CoV-2) cause respiratory illnesses of varying severity, from the common cold to fatal pneumonia [4, 5]. Before SARS appeared, coronaviruses had never been particularly dangerous to humans, causing severe diseases only in animals [6].

According to the World Health Organization (WHO), the onset of the SARS epidemic occurred in Guangdong, China, in November 2002, followed by the worldwide spread of the virus with reported cases in 29 countries, including Canada and the USA. However, 8 months later, in July 2003, after causing 774 deaths, the SARS epidemic was declared to be contained by the WHO [7].

Only a decade later, another pathogenic coronavirus, MERS-CoV, caused an endemic in Middle Eastern countries. Since 2012, there have been at least 845 MERS-CoV-related deaths in 27 countries but about 80% of the reported cases were in Saudi Arabia. Nowadays, it continues to cause sporadic outbreaks, mostly localized in the Arabian Peninsula [8].

The outbreak of the novel highly contagious SARS-CoV-2 was first identified in Wuhan, Hubei province, China, in early December 2019 [9]. The SARS-CoV-2 virus rapidly spreads across continents and on 30 January 2020, the WHO declared the outbreak of a Public Health Emergency of International Concern and a pandemic on 11 March. Infections with SARS-CoV-2 are now widespread, and as of 31 August 2020, 25,467.390 cases have been confirmed in more than 110 countries, with 851,000 deaths [10–13].

The three Coronaviruses belong to the same *Betacoronavirus* genus [14]. Clinical presentation of COVID-19, the disease caused by SARS-CoV-2 virus, shows great similarities with SARS and MERS pneumonia. More than 80% of cases are mild, and patients normally recover within 2 weeks. However, some patients show severe symptoms, like acute respiratory distress syndrome (ARDS) and about 5% show critical conditions, which may evolve into septic shock or multiple organ failure [13].

According to early phylogenetic studies, SARS-CoV-2 is related to SARS and both of them show over 85% genome sequence identity with the bat SARS-like CoV, which would suggest the bat origin of the virus [15, 16].

Several studies hypothesized the entry of these three viruses in humans from their natural reservoir bats, via intermediate host like civets and camels, in the case of SARS-CoV and MERS-CoV, respectively. The intermediate host of the SARS-CoV-2 still needs to be established, although some studies suggest pangolins as a possible host [17].

Although SARS and MERS have significantly higher mortality rates than COVID-19, the novel SARS-CoV-2 is more infectious and the overall number of deaths from COVID-19 far outweighs that from SARS or MERS [18–20]. Table 1 summarises the main features of the three coronaviruses.

**Table 1:** Comparative analysis of the main features of SARS-CoV, MERS-CoV and SARS-CoV-2

Disease	Pathogen	Family	Origin	Intermediate	Mortality rate	R_0_	Total number of deaths
SARS	SARS-CoV	β coronaviridiae	Bats	Civets	9.5%	1.8	774
MERS	MERS-CoV	β coronaviridiae	Bats	Camels	34.4%	0.7	858
COVID-19	SARS-CoV-2	β coronaviridiae	Bats	Pangolins	2.3%	2.0–2.5	> 850,000

Countries are racing to slow the spread of the virus by testing and treating patients, carrying out contact tracing, limiting travel, quarantining citizens, and cancelling large gatherings such as sporting events, concerts and schools [21].

Early diagnostic tests are essential tools to track the spread of the virus in order to control the epidemic. At the moment, most testing for COVID-19 is currently done on viral genetic material from nasopharyngeal swabs, using the reverse transcription polymerase chain reaction (RT-PCR), a molecular biology technique which amplifies a specific genetic sequence of the virus. Alternatively, the enzyme-linked immunosorbent assay (ELISA), a common biochemical technique, is used to detect the specific antibodies or antigens in patient blood. Unfortunately, both methods require expensive instruments and the use of specialized laboratories and well-trained personnel [22]. In particular, serological tests show evidence of low sensitivity, accuracy and specificity [23].

Obviously, the first method tells if a person is currently infected, whereas the second method can determine if a patient has at some point been infected by SARS-CoV-2. Reverse transcription loop-mediated isothermal amplification (RT-LAMP) is a more recent molecular technique, where the amplification is conducted at a single temperature and does not need specialized laboratory equipment [24]. However, all these methods are not suitable for point-of-care testing [11, 25].

Therefore, rapid, accurate, low-cost, miniaturized diagnostic platforms for virus detection usable at the point-of-care remain a challenge [25, 26]. Lateral flow immunoassays are qualitative chromatographic assays, similar to common pregnancy tests, based on a two-site non-competitive format, as ELISA tests, but usable at the point-of-care. They are produced as test kits to be used by a specialist or by the patients themselves, but they suffer from a poor sensitivity [27]. The main characteristics of current methods for COVID-19 detection are summarized in Table 2.

**Table 2:** Main characteristics of current methods for COVID-19 detection

Method	Biomarker	Lab or POC	Sample site	Time of analysis
RT-PCR	Viral RNA	Lab based	Nasopharyngeal swab	~ 4 h
LAMP	Viral RNA	Lab based	Nasopharyngeal swab	~ 3 h
Lateral flow	Antibody (or antigen)	POC	Blood	~ 15 min
ELISA	Antibody (or antigen)	Lab based	Blood	~ 2 h

Affinity-based biosensors (ABBs) represent interesting diagnostic tools for early and affordable detection of virus diseases, thanks to their properties, such as high sensitivity, high specificity, fast response time and the possibility of miniaturization for POC use [22, 28–31]. These peculiar characteristics allow them to complement current methods of screening and monitoring of a virus outbreak, especially when in situ and real-time analysis is required.

The recent advances in nanotechnology and the use of nanomaterials in the construction of biosensors resulted in a significant improvement in the performances of these devices [32–34]. Nanomaterials allowed a large increase in biosensors efficiency and sensitivity, thanks to their excellent conductivity, extraordinary photoelectrochemical properties and the possibility of miniaturization of the sensing platform [35–39].

In this review, we describe the nanobiosensors based on affinity interactions reported in the literature for the detection of SARS, MERS and COVID-19. A comparison with non-nanostructured affinity-based biosensors and with conventional methods is also provided. The review is structured into four sections. The first section presents a brief overview of the different classes of nanomaterials used for affinity-based biosensors purposes, and the other sections are divided by analyte type, with a particular focus on COVID-19, because of the urgent need to find early diagnostic methods for SARS-CoV-2 detection, in order to deal with the current COVID-19 pandemic.

## Affinity-based nanobiosensors

The use of novel nanomaterials in biosensing may overcome some of the challenges and limitations of biosensor technology. Nanomaterials by definition must have dimensions in the range 1–100 nm. They are developed to exhibit novel characteristics compared to the same material without nanoscale features, such as increased strength, conductivity and unique optical, magnetic, thermal and chemical properties.

Affinity-based nanobiosensors combine the high specificity of the biorecognition agents, namely bioreceptors, such as antibodies, ssDNA and aptamers, with the extraordinary properties of the nanomaterials, which allow enhanced sensitivities and lowered detection limits of several orders of magnitudes [37, 40].

The high specific surface interaction of the nanobiosensor with the bioanalyte becomes highly efficient thanks to the extremely large surface/volume ratio, which enables the immobilization of an enhanced amount of bioreceptor units. Thus, the immobilization strategies used to conjugate the biorecognition agents onto the nanomaterials remain a constant challenge [41]. The technique used for the bioreceptor immobilization is one of the key factors in developing a reliable affinity-based nanobiosensor. In addition to the immobilization of the bio-molecules, the nanomaterials can serve for target recognition and for signal transduction and amplification [40].

Various kinds of nanomaterials have been merged to ABBs, such as noble metal nanoparticles, carbon nanostructures, quantum dots and magnetic nanoparticles [34, 35, 42–44].

**Fig. 1:**
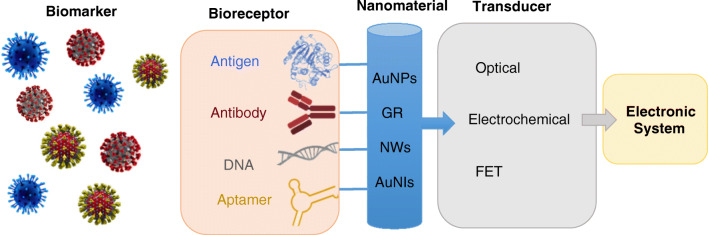
Schematic diagram of nanomaterial-based affinity biosensor for coronavirus detection. *List of abbreviations*: AuNPs, gold nanoparticles; GR, graphene; NWs, nanowires; AuNIs, gold nanoislands; FET, field effect transistor

The ABBs for coronavirus detection reported in the literature are based on gold nanoparticles (AuNPs) and nanoislands (AuNIs), graphene (GR), and nanowires (NWs). Figure 1 shows a schematic diagram of a nanomaterial-based affinity biosensor for coronavirus detection.

### Gold nanoparticle and gold nanoisland affinity-based biosensor

Among the group of metal noble nanoparticles (MNPs), gold nanoparticles (AuNPs) are mostly used in biosensing application for virus infections due to their outstanding optical/electrical properties, excellent biocompatibility, catalytic properties and relatively simple production pathway [45–47]. MNPs have been widely used as supporting electrode materials increasing electron transfer rates and surface-to-volume ratio, thus allowing the immobilization of large amounts of primary antibodies or cDNA, suppressing the non-specific binding (NSB) of proteins, with a consequent enhancement of the analytical response of the device.

AuNPs play a different role in the biosensing process depending on the transduction mode of the biosensor, namely optical and electrochemical biosensors. There are several optical sensing modalities for AuNPs, surface plasmon resonance (SPR) being the one that attracted most intensive research, as AuNPs are considered to have the ability to amplify the SPR signal [48]. As for the electrochemical biosensors, AuNPs allow to improve the analytical performances of the device through a double mechanism: (i) as novel immobilization platforms/electrochemical transducers, which allow loading of a larger amount of the biosensing element, thanks to the much higher surface area of AuNPs compared to flat gold surfaces; (ii) as labels for signal amplification [40].

AuNPs are often conjugated with other nanomaterials to further improve their binding capacity. In this context, carbon nanotubes (CNTs) have attracted much interest due to their unique properties [49]. Nanohybrids of AuNPs and CNTs have been realized, offering a more effective immobilization matrix. Platforms based on gold nanoislands were also used for numerous sensing applications [50]. They are basically gold aggregates with dimensions in the range 20–80 nm, obtained by deposition and annealing of the AuNPs at high temperature (560 °C) for several hours (~ 10 h).

### Graphene affinity-based biosensors

Graphene (G), together with carbon nanotubes (CNTs), is the most promising nanostructured carbon materials for biosensing applications, where each allotrope has its own specific properties and advantages as a transducer element. Carbon nanotubes are one-atom-thick sheets of graphite, called graphene, rolled into cylinders with a diameter of the order of a few nanometres [51–58]. Compared to CNTs, graphene has a much younger history. Graphene and CNTs share some properties in common, including excellent mechanic, electronic and thermal properties [59–61]. Thanks to its defect-rich property, graphene can be easily functionalized by inserting functional groups on the 2D plane, thus becoming a good support for immobilizing of ligands, such as antibodies and single-strand DNA for developing immunosensors and aptasensors, respectively [43, 62, 63]. Graphene, as well as CNTs, is water insoluble, which can be overcome by modifying its surface with hydrophilic functional groups in order to increase its solubility and suppress the NSB of proteins onto the electrode surface. Another common strategy to minimize NSB is achieved by graphene functionalization through its oxidation to graphene oxide (GO) and reduced graphene oxide (rGO), which found a lot of applications in immunosensors development [64]. The choice between the use of G or GO depends on the need of functionalization of the nanocarbon for biomolecule immobilization (more oxygen groups required) or of a higher conductivity (less oxygen groups required).

As for CNTs, graphene is largely used in electrochemical and optical biosensors or field-effect transistor (FET) setups, where the changes in the conductivity of the graphene channel after the biorecognition event led to high sensitivities [59, 65]. Contrary to CNTs, ssDNA or oligonucleotide bioreceptors are reversibly adsorbed to graphene oxide and successively released after the recognition event, thus allowing the recovering of the bioreceptors [34].

### Nanowire affinity-based biosensors

Nanowires (NWs) are one-dimensional nanostructures in the form of wire that can be composed of both metallic and non-metallic elements with nanometre sized diameters and micron long lengths. The NWs are robust and have high physical strength, directly attributed to their crystalline structure, and unique 1D morphology, electrical, mechanical, optical, magnetic and thermal properties. Silica NWs are broadly explored for biosensor applications, thanks to their optical, photonic and electronic properties with excellent biocompatibility for sensing application [66, 67].

Silicon and indium oxide NWs are mostly explored as novel biosensing tools for highly sensitive virus detection, due to their wide bandgap, which broadens the scope of detection from purely electrochemical or FET-based detection to more simple optical methods [68, 69].

## Nanobiosensors for SARS detection

**Fig. 2:**
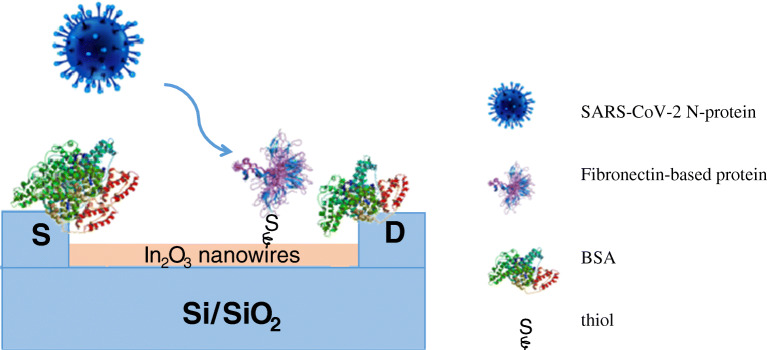
Schematic representation of In_2_O_3_ nanowires FET-based immunosensor for SARS-CoV*. List of abbreviations*: S, source; D, drain; BSA, bovine serum albumin

Up to now, there is only one nanobiosensor for SARS-CoV detection reported in the literature. It is a FET-based immunosensor, developed by Ishikawa and coworkers in 2009 [70], where the antigen-antibody binding generates a change in conductance, correlated to the virus concentration. The biomarker used for SARS-CoV detection is the virus antigen nucleocapsid protein (N-protein), the most abundant protein in coronaviruses. Instead of conventional antibodies, antibody mimic proteins (AMPs) have been utilized as bioreceptors. The AMPs can be easily produced by in vitro selection techniques and are smaller and stable at a wider range of pH than normal antibodies. A fibronectin-based protein (Fn) has been properly engineered as AMP capture agent. The exposed gate region of the FET-based immunosensor was modified with In_2_O_3_ nanowires on a Si/SiO_2_ substrate, in order to improve the immobilization of the AMPs and the signal transducing. The Fn protein was anchored to the NWs via the only thiol functional group present in the whole peptide sequence from a cysteine residue. The schematic diagram showing the covalent immobilization of the Fn probe onto the nanowires is represented in Fig. 2. At the working pH = 7.4, the N-proteins are positively charged and therefore their binding on a p-type channel causes depletion of charge carriers (holes) and a consequent decrease in conductance. Bovine serum albumin (BSA) was used as a “blocking agent” for nanowires and source-drain electrodes, thus avoiding NSB, which may lead to false-positive results (Fig. 2). The so-developed platform was able to detect the N-protein at sub-nanomolar concentrations, in the presence of 44 μM BSA, with a comparable sensitivity to current immunological detection methods, but with a shorter detection time and without the need of labelled reagents. Indeed, the high sensitivity and high selectivity of the proposed biosensor are achieved by the synergic effect of the In_2_O_3_ nanowires/Fn protein, which is able to selectively detect the SARS biomarker N-protein. Moreover, compared to other metal oxide nanowires (ZnO, SnO_2_, etc.), In_2_O_3_ nanowires have the advantage that they do not possess an insulating oxide layer, such as SiO_2_ for Si nanowires, which can decrease the sensor sensitivity, and therefore, they contribute to a marked decrease of the detection limit of the sensor.

Table 3 shows the characteristics and the analytical performances of the described SARS immunosensor and a comparison with other “non-nanotechnology-based” immunosensors for SARS detection, reported in the literature. In particular, two SPR-based biosensors [71, 72] and one piezoelectric biosensor [73] have been realized but their sensitivities resulted to be lower of several orders of magnitudes.

**Table 3:** Upper part: affinity nanobiosensors for SARS, MERS and COVID-19 detection. Lower part: comparison with non-nanostructured biosensors reported in literature for coronavirus detection

Disease	ABBs type	Transducer	Biomarker	Biosensor Platform	Biosensor format	Linear range	LOD	References
SARS	Immunosensor	FET	Antigen N-protein	Si-SiO_2_/In_2_O_3_ nanowires/AMP fibronectin	Label-free	-	SubnM	[70]
MERS	Immunosensor	Amperometric	Antigen spike protein S1	AuNPs/MERS-CoV antigen/MERS-CoV Ab	Label-based competitive	0.001–100 ng/mL	0.4 pg/mL	[74]
COVID-19	Immunosensor	FET	Antigen spike protein S1	Si-SiO_2_/graphene/SARS-CoV-2 Ab	Label free	-	1 fg/mL	[75]
COVID-19	DNA-sensor	PPT+LSPR	SARS-CoV-2 nucleid acid	AuNIs/cDNA	Label free	0.1 pM −1 mM	0.22 pM	[76]
SARS	Immunosensor	SPR	Anti-SCVme Ab	Au/GBP-SCVme antigen	Label free	-	200 ng/mL	[77]
SARS	Immunosensor	SPR	Antigen N-protein	SARS-CoV Ab	Label-based sandwich	0.001–10 ng/mL	1 pg/mL	[78]
SARS	Immunosensor	Piezoelectric	SARS-CoV antigen	PQC/SARS-CoV Ab	Label free	0.6–4 μg/mL	-	[79]

*List of abbreviations*: *Ab*, antibody; *AMP*, antibody mimics proteins; *SCVme*, SARS-CoV membrane-envelope protein; *GBP*, gold binding polypeptide; *PQC*, piezoelectric quartz crystal; *PPT*, plasmonic photothermal localized SPR; *AuNIs*, gold nanoislands; *cDNA*, complementary DNA

## Nanobiosensors for MERS detection

An amperometric nano-immunosensor for MERS-CoV virus detection was described in 2019 by Layqah and Eissa [74]. In this case, the virus spike protein S1, which is the common target for neutralizing antibodies, was utilized as MERS biomarker [80, 81].

**Fig. 3:**
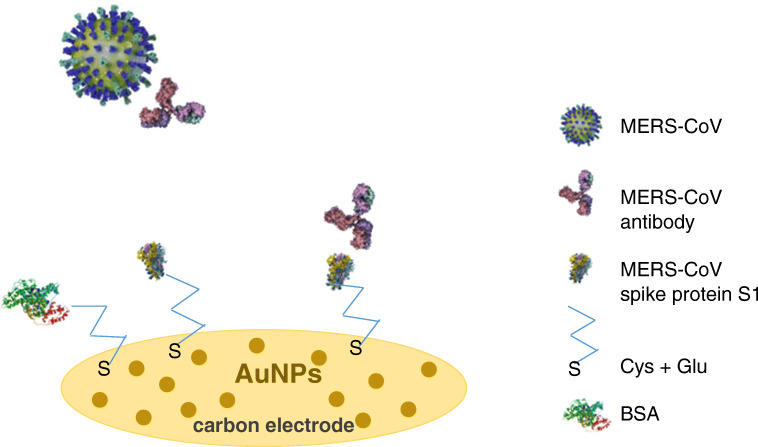
Schematic representation of AuNPs immunosensor for MERS-CoV. *List of abbreviations*: Cys, cysteamine; Glu, glutaraldehyde; BSA, bovine serum albumin

The biosensor is based on an indirect competition between the free virus in the sample and immobilized MERS-CoV recombinant spike protein S1, for a fixed antibody concentration added to the sample. The immunosensor was realized on an array electrodes system, thus allowing the simultaneous detection of MERS-CoV and HCoV, another human coronavirus. The surface of the carbon electrodes was modified with AuNPs, in order to enhance the electrochemical properties of the electrode, providing a higher surface area and a faster electron transfer rate. Successively, MERS-CoV and HCoV antigens were immobilized onto the AuNPs/carbon electrode, by a simple drop-casting procedure, after incubating the electrode in a solution containing cysteamine and glutaraldeyde, for covalently binding of the NH_2_ groups of the antigens. A schematic representation of the AuNPs immunosensor is shown in Fig. 3. The non-specific adsorptions were reduced by incubating the electrode in BSA solution, in order to block the unreacted aldehyde groups and the free gold surface. The experimental conditions were carefully optimized, in particular the concentration of antibody used for incubation of the antigen-modified electrode and the binding time, resulting in 10 μg/mL and 20 min, respectively. The detection was obtained by measuring the peak current signal of the ferro/ferricyanide redox probe, properly added to the solution, with the square wave voltammetry (SWV) technique. A decrease of the SWV peak current is clearly observed after binding of the antibodies to the immobilized antigens, because of the “coverage” of the electrode surface by the antibody molecules. Thus, a decrease of both electron transfer efficiency and current is registered. The so-realized immunosensor showed a good linear response from 0.001 to 100 ng/mL for MERS-CoV and a very high sensitivity, with a detection limit of 0.4 pg/mL, definitely lower value than that obtained with ELISA method (1 ng/mL) [82]. The characteristics of the biosensor are summarized in Table 3. The selectivity of the biosensor was studied by using different virus proteins, such as FluA and FluB, showing no cross-reactivity phenomena. The possibility of the use of the proposed biosensor for simultaneous detection of different types of CoVs was also confirmed by mixing the two proteins MERS-CoV and HCoV on the electrode surface. The stability of the sensor was good, as the sensor showed only 2% current decrease after 2 weeks. Finally, the proposed immunosensor was successfully tested in spiked nasal samples showing good recovery percentages.

## Nanobiosensors for COVID-19 detection

### Viral antigen detection

**Fig. 4:**
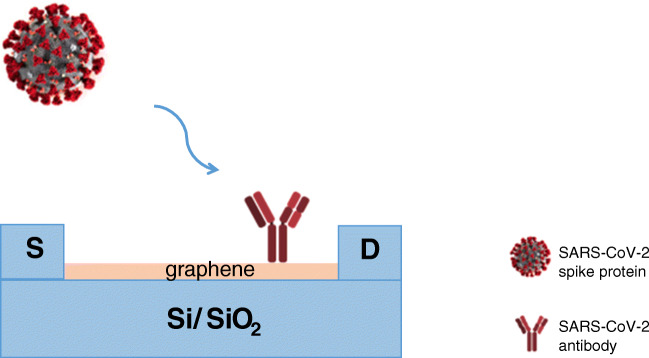
Schematic representation of graphene FET-based immunosensor for SARS-CoV-2. *List of abbreviations*: S, source; D, drain

The first biosensing strategy is the use of antibodies or cDNA to selectively capture the viral antigen or viral RNA. A graphene-based FET device has been properly engineered by Seo and coworkers to determine SARS-CoV-2 viral load in nasopharyngeal swabs of patients affected by COVID-19, thus allowing also the determination of the severity of the COVID-19 disease [75]. The sensing area of the FET-based biosensor is a graphene sheet, transferred to a SiO_2_/Si substrate, and successively modified with SARS-CoV-2 spike antibody, properly immobilized onto the graphene sheet surface by drop-casting, as schematized in Fig. 4. The device allowed the detection of the SARS-CoV-2 antigen spike protein at concentrations as low as 1 fg/mL in phosphate buffer, a value much lower than that reported with ELISA and PCR methods [83]. The biosensor was tested in the universal transport medium (UTM), used for suspending the nasopharyngeal swabs for real clinical analysis. No reagent contained in UTM affected the measurements and the detection limit resulted to be 100 fg/mL. In addition, the proposed COVID-19 sensor showed no significant response to MERS-CoV spike proteins, assuring high selectivity and specificity for the SARS-CoV-2 spike antigen protein. Finally, the performance of the sensor was tested in real clinical samples, collecting nasopharyngeal swab specimens of COVID-19 patients and of normal subjects. The COVID-19 FET-based nanobiosensor allowed to discriminate between patient and normal samples with detection limits lower than those reported with other current methods, without any sample preparation or preprocessing.

**Fig. 5:**
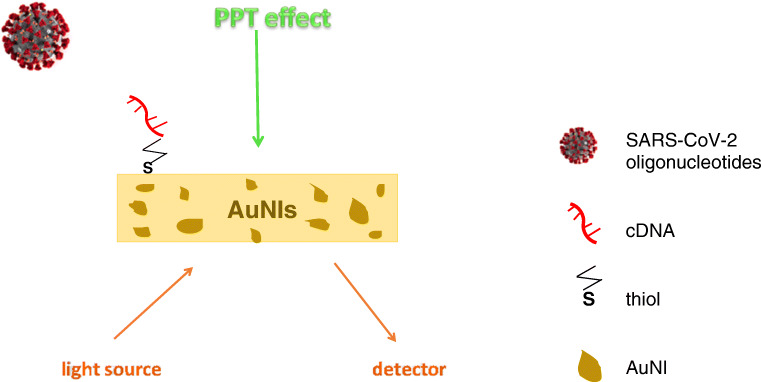
Schematic representation of AuNIs PPT-enhanced LSPR DNA-sensor. *List of abbreviations*: AuNIs, gold nanoislands; PPT, plasmonic photothermal; LSPR, localized surface plasmon resonance; cDNA, complementary DNA

Noble metal nanostructures have been frequently used for virus detection systems to enhance functions such as specificity and sensitivity. A DNA-nanosensor for SARS-CoV-2 detection was recently proposed by Qiu and coworkers [76]. They realized a dual DNA-sensor consisting of a single chip, modified with a two-dimensional distribution of gold nanoislands (AuNIs). The chip integrates the plasmonic photothermal (PPT) effect and the localized surface plasmon resonance (LSPR) sensing transduction. The sensor chip was functionalized with complementary DNA (cDNA) receptors by forming Au-S bonds between the AuNIs and the thiolic groups of cDNA. A schematic representation of the AuNIs PPT enhanced LSPR biosensor is shown in Fig. 5. The proper surface functionalization can suppress the non-specific binding events, thus increasing the sensitivity of the biosensor. The PPT heat, generated in situ on the same AuNI chip when illuminated at their plasmonic resonance frequency, was capable to significantly improve the kinetics and the specificity of the hybridization of SARS-CoV-2 nucleic acid sequences to their cDNA. A large number of false positive or false negative have been reported with current methods of COVID-19 detection. The PPT heating is capable of inhibiting the spurious binding of non-matching sequences, thus avoiding an incorrect diagnosis. The dual-functional biosensor exhibited a linear range between 0.1 pM and 1 mM with a detection limit of 0.22 pM, which resulted low enough for direct analysis of SARS-CoV-2 sequences in respiratory real samples. Similar multiple non-specific gene sequences from SARS-CoV and SARS-CoV-2 were tested and discriminated, attesting the high selectivity of the biosensor towards cross-reactive and interfering sequences.

The analytical performances of the reported COVID-19 nanobiosensors are summarized in Table 3.

Another promising tool for detection of SARS-CoV-2 viral genome is the Clustered Regularly Interspaced Short Palindromic Repeats (CRISP)–associated (Cas) enzymes technology, which allowed the detection of specific COVID-19 gene sequences with detection limits between 10 and 100 copies per microliter in less than 1 h employing a target amplification (RPA) [84]. Recently, a research group has announced his interest to develop an amplification free electrochemical CRISP biosensor for on-site COVID-19 testing, with nanomodification of the electrode surface for signal enhancement [71].

Another recent study describes a portable graphene-based electrochemical biosensor for highly sensitive POC testing of *Vibrio parahaemolyticus* in seafood, which could be translated for COVID-19 detection. The detection was carried out using loop-mediated isothermal amplification (LAMP) and a graphene-based screen-printed electrode (SPE). The interaction between SPE and amplicons results in a shift in cathodic current, which stems from the intercalation of redox probe to double-stranded DNA. A portable mini potentiostat is used with SPE for on-site POC detection [85]. Most recently, PathSensors Inc. announced the development of a “Canary” fast biosensor for SARS-CoV-2 aerosol detection. The proposed platform utilizes a cell-based immunosensor that couples capture of the virus with signal amplification and provides a result in 3–5 min. PathSensors is based on a genetically engineered immune cell able to identify and bind to a specific target pathogen and then light up when the target pathogen is found. By measuring the light output from the cell, it is possible to know if the target pathogen is present in the sample. The initial application of the PathSensors device will be for testing of environmental swabs and air monitoring in sensitive spaces, such as hospitals, offices and food services. Validation data of the new biosensors will be available soon [72].

### Antibody detection

A different approach in the detection of COVID-19 infection is the development of nanobiosensors for *anti*-SARS-CoV-2 detection, after functionalization of the sensor’s surface with the specific viral antigens.

Serologic assays for SARS-CoV-2 antibodies are now broadly available and play an important role in understanding the virus epidemiology in the general population and identifying groups at higher risk for infection. Unlike viral direct detection methods that can detect acutely infected persons, antibody tests are indirect tests that help determine whether the individual being tested was ever infected, even if that person never showed symptoms, by measuring the host humoral immune response to the virus. Therefore, serology antibody assays do not typically replace direct detection methods as the primary tool for diagnosing an active SARS-CoV-2 infection, but they do have several important applications in monitoring and responding to the COVID-19 pandemic. Demographic and geographic patterns of serologic antibody test results can help determine which communities may have experienced a higher infection rate and therefore may have higher rates of herd immunity. Moreover, serologic test results may assist with identifying persons potentially infected with SARS-CoV-2 and determining who may donate blood that can be used to manufacture convalescent plasma, as a possible treatment for COVID-19 disease.

Like infections with other pathogens, SARS-CoV-2 infection elicits the development of IgM, IgA and IgG antibodies. IgA and IgM reach their peak during 4–25 days after illness onset, whereas IgG during 21–25 days, and therefore, they are used for diagnosis at early and late stages, respectively [73]. Anyhow, it remains uncertain how long the immunoglobulins remain detectable following infection and whether individuals with antibodies are protected against reinfection with SARS-CoV-2.

At present, no immuno- or DNA-sensors for the detection of immunoglubulins against SARS-CoV-2 are available on the market. Several nanobiosensors for the detection of specific antibodies against viral antigens or biomarkers have been extensively explored in literature, which could potentially being used for COVID-19 [86–88]. For example, a recent study has reported the development of a label-free electrochemical biosensor with an aptamer-functionalized black phosphorus (BP) nanostructured electrode [89]. The BP nanosheets are functionalized with *anti*-Ab-aptamers after coating with poly-L-lysine (PLL). BP-based biosensors show higher detection sensitivity and specificity compared to reduced graphene oxide biosensors, achieving detection limits down to pg level and ng level, respectively. A similar platform could be used also for highly sensitive detection of IgG or IgM against SARS-CoV-2 in patient blood samples.

### IoT biosensors

An important issue regarding the use of nanobiosensors for early POC diagnosis and prevention of COVID-19 is their capability to upload collected data via a Bluetooth interface to an Android-based smartphone, which will successively transfer them to Health Centres Authorities to tackle the disease spread, as already realized by Zhou et al. [90], for an IoT (Internet of Things) real-time PCR system for dengue fever virus spread control.

The IoT applied to medicine, also called the Internet of Healthcare Things (IoHT), encompasses medical devices and software applications connected to the Internet, offering extensive healthcare services. The IoT has opened up a world of possibilities in the medical field: when connected to the Internet, ordinary medical devices can collect invaluable additional data, give extra insight into symptoms and trends, enable remote care, track medication orders and use wearable devices to transmit health information to the concerned health care professionals [91]. Future studies should further improve the function of smartphone and specific smartphone apps to enable on-site data analysis while allowing data storage to track patient health status.

**Fig. 6:**
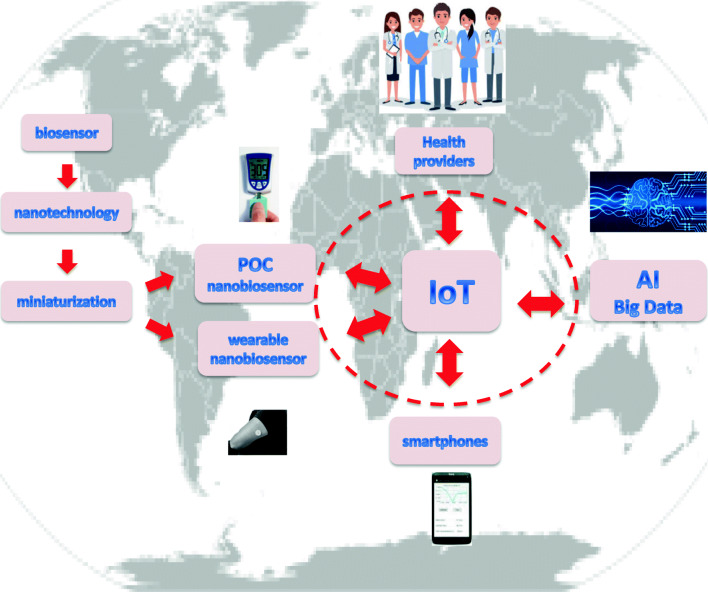
Next-generation IoT nanobiosensor-based diagnostics system

By combining biosensors, artificial intelligence (AI), information technology and dynamic networking devices, IoT could provide long-distance communication between nanobiosensors, hospitals and patients, thus improving current medical conditions [92]. In Fig. 6 is a schematized IoT architecture of a next-generation nanobiosensor-based diagnostics system.

At present, an Internet of Things (IoT) based on a functional POC instrument for SARS-CoV-2 antibody detection is not available. The POC-measured biosensors data could be automatically uploaded via Bluetooth to the patient’s smartphone or tablet and then sent through global communications to a central epidemiological data centre, for automated monitoring of the epidemiological situation. Obtained data could be made ready to fit into epidemiological models in order to forecast evolution of the epidemic outbreak. Suitable modelling algorithms have already been developed through AI to predict key parameters for clinical practitioner, polling IgG and IgM test results, to provide advanced diagnosis of individuals, to characterize the own progress of the illness and to help in categorizing patients, especially in the “transition zones”, where the decision can be more doubtful (Fig. 6). Each validated diagnosis instance of a given patient, once anonymized, could be automatically transmitted to a central data station, for real-time advanced monitoring of the epidemics, epidemiological management, prevention of new virus outbreaks and evaluation of success of eventual vaccination. Recently, several contact tracing apps have been developed, thus offering technological solutions to the problem of controlling the spread of the virus and have worked reasonably well in countries such as Singapore, China, South Korea and other parts of Asia. However, in other countries, privacy concerns are limiting their introduction, which could limit efforts to control the pandemic [93].

## Conclusions and future perspectives

The incorporation of nanomaterials into affinity-based biosensing applications has successfully demonstrated to allow fast, sensitive and reliable detection of SARS, MERS and COVID-19. In particular, nanomaterial-based FET biosensors empowered the achievement of elevated biosensor performances, in terms of high sensitivity, selectivity and low detection limits. Graphene and In_2_O_3_ nanowires have been used for the exposed gated micro-region of the FET for very high electrochemical detection of SARS-CoV-2 and SARS-CoV, respectively. Other nanomaterials, such as gold nanoparticles and gold nanoislands, were successfully employed for the development of an immunosensor and a DNA-sensor for MERS and COVID-19, respectively, with detection limits in the fempto-pico molar range.

An important feature of the nanobiosensors with electrochemical and FET transduction is the possibility of their miniaturization into inexpensive and integrated platforms, similar in operation to handheld electrochemical readers, usable for POC diagnostics. Additionally, the affinity biosensors can be easily multiplexed, by incorporating multiple individually accessible electrodes on the same platform, thus allowing simultaneous detections. Of course, further efforts will be required before miniaturized POC multiplex testing practical applications are feasible.

Despite these encouraging properties, very few examples of nanobiosensors for SARS, MERS and COVID-19 detection have been developed and successfully applied in real clinical analysis so far. Of course, a lot of studies for COVID-19 are currently in progress. Point-of-care nanosensors for detection of SARS-CoV-2 antibodies are under development. At the moment, only lateral flow immunoassays produced by different companies are commercially available as single-use POC tests for *anti*-SARS-CoV-2 antibodies [94]. They are paper-like membrane strips coated with gold nanoparticle-antigen conjugates in the conjugation pad and antigens in the nitrocellulose membrane. A blood drop of the patient is deposited on the sample pad and is moved across the test by capillary action. Immobilized antibodies recognize and bind all human IgG and IgM. However, only human IgG-IgM/gold nanoparticle-antigen conjugates will produce a visible coloured line.

A next issue in nanobiosensing design should be the integration of the extraction system into the proposed biosensor to make it wearable and user-friendly. One possibility would be to translate the developed platforms into microneedles-based biosensors, which allow a continuous monitoring of SARS-CoV-2 antigens, antibodies or nucleic acid in the dermal interstitial fluid of both symptomatic and asymptomatic population. These wearable devices would provide important information on the following: (i) prevalence of infection in the community; (ii) development and decay of immunity in a population (the dynamics of “herd” immunity). As with individuals, the levels of antibody change over time (whether from natural infection or vaccination) and continuous measurements allow this to be tracked and potentially predict the likelihood of successive waves of the pandemic; (iii) post vaccination immune response, possibly indicating the need for a “booster” vaccination.

In summary, significant challenges are yet to be addressed and a lot of effort is worth to be invested in future studies for the development of IoT wearable nanobiosensors for COVID-19 detection, thanks to their great potential to perform rapid, accurate and in situ early diagnosis and, more importantly, to track the infectious diseases, thus preventing further pandemic outbreaks.
